# Resampling-based evaluation of a 79-SNP panel for hybrid classification across generations: a case study in European lobster (*Homarus gammarus*)

**DOI:** 10.1093/g3journal/jkag206

**Published:** 2026-07-29

**Authors:** Erik Sandertun Røed, Charlie D Ellis, Jamie R Stevens, Louise Chavarie, Marie Saitou

**Affiliations:** Centre for Ecological and Evolutionary Synthesis, Department of Biosciences, University of Oslo, Blindernveien 31, 0371 Oslo, Oslo, Norway; Faculty of Biosciences, Norwegian University of Life Sciences (NMBU), Oluf Thesens vei 6, 1433 Ås, Akershus, Norway; Department of Biosciences, University of Exeter, Prince of Wales Road, Exeter, Devon EX4 4PS, United Kingdom; Department of Biosciences, University of Exeter, Prince of Wales Road, Exeter, Devon EX4 4PS, United Kingdom; Faculty of Environmental Sciences and Natural Resource Management, Norwegian University of Life Sciences (NMBU), Høgskoleveien 12, 1433 Ås, Akershus, Norway; Faculty of Biosciences, Norwegian University of Life Sciences (NMBU), Oluf Thesens vei 6, 1433 Ås, Akershus, Norway

**Keywords:** hybridization, introgression, SNP panel, conservation genetics, fishery management

## Abstract

Accurate detection of hybridization and introgression is critical not only for basic research, but also for fisheries management. In many species, however, hybrid ancestry is difficult to detect beyond the F1 generation, especially when based on limited genetic markers. In European waters, hybridization between the native *Homarus gammarus* and the introduced *H. americanus* poses a direct risk to the integrity of native stocks and effective fishery management, yet detection methods are often limited to morphological traits or first-generation hybrids. A set of 79 SNPs originally developed for population genetic analyzes in *H. gammarus* and subsequently applied to distinguish *H. gammarus*, *H. americanus*, and F1 individuals has shown promise, but its capacity to resolve later-generation backcrosses remains untested. Here, we evaluated this panel's performance under the allele-frequency distributions and marker properties observed in the empirical dataset it was based on, using individual-based resampling to generate genotypes with known ancestry proportions. We show that the panel retains discriminatory power for broad ancestry classes and early-generation hybrids, but with reduced accuracy for later-generation backcrosses and especially high uncertainty in distinguishing backcrosses toward *H. gammarus* from variation within the *H. gammarus* parental reference. These findings provide an assessment of hybrid classification performance for this specific panel and demonstrate the value of empirical resampling simulations for identifying both the utility and the limitations of existing marker panels in fisheries-relevant species.

## Introduction

Accurate identification of species boundaries is essential for understanding gene flow, reproductive isolation, and the ecological consequences of admixture (Chan et al. 2017; Christie and Strauss 2019; Satokangas et al. 2023; Wu et al. 2024; Chambers et al. 2025). In many taxa, however, species boundaries are obscured by cryptic genetic divergence or by hybridization, particularly following secondary contact between closely related species (Sardell and Uy 2016; Porto-Hannes et al. 2021; Termignoni-Garcia et al. 2022; Peng et al. 2025). Cryptic divergence, the lack of morphological differentiation between genetically distinct lineages, complicates taxonomic classification and biodiversity assessment (Struck et al. 2018; Olivares et al. 2024; Peng et al. 2025), while hybridization can generate admixed individuals whose phenotypes overlap extensively with those of parental species (Antoniou et al. 2018). Molecular tools that quantify ancestry gradients are therefore needed to delineate species identity and levels of introgression in natural populations (Frade et al. 2010; Valencia-Montoya et al. 2020; Massatti and Winkler 2022; Satokangas et al. 2023; Yuan et al. 2025).

One such system is the European lobster (*Homarus gammarus*), which is increasingly threatened by hybridization with the introduced American lobster (*Homarus americanus*) (Jørstad et al. 2011; Davies and Wootton 2018; Barrett et al. 2020). The introduced *H. americanus*, which is typically larger and more aggressive than the native *H. gammarus*, poses multiple risks to European lobster populations, including direct competition, predation, and the potential transmission of novel pathogens (Jørstad et al. 2011). Such risks may further exacerbate the regional decline of *H. gammarus*, a red-listed species in parts of its range, by reducing population viability and resilience (Kleiven et al. 2012; Marcussen et al. 2025). In addition, hybridization with *H. americanus* raises concerns about maladaptive introgression and the long-term genetic integrity of native populations (Todesco et al. 2016).

Records of *H. americanus* in European waters are generally interpreted as resulting from repeated introductions via escape or deliberate release from the live seafood trade, rather than from natural range expansion or established self-sustaining populations (Spurr 1974; Jørstad et al. 2007; Galil et al. 2011). Most records are of adult *H. americanus* observed in European waters after accidental or illegal releases, together with sporadic reports of hybrid individuals. Notably, evidence of interspecific mating and hybrid fertilization has been documented in the wild: Ellis et al. (2020) analyzed hybrid larvae hatched from a wild-caught ovigerous *H. americanus* female captured in Sweden. However, these larvae hatched and were sampled in a rearing facility rather than in wild waters, and evidence for successful recruitment of americanus-origin juveniles in Europe remains limited, with the only reliable juvenile record being a single American lobster caught off the northeast coast of England (Tinlin-Mackenzie et al. 2022). The temporal scale of hybridization in the wild remains unclear. There is currently no robust evidence for the number of hybrid generations or the presence of stable introgressed populations.

Morphological traits commonly used to identify *H. americanus* or putative hybrids in European waters are not consistently diagnostic, particularly for admixed individuals and backcrosses (Jørstad et al. 2007, 2011; Stebbing et al. 2012). As a result, genetic tools have become central to monitoring the spread of non-native individuals and assessing the extent of hybridization. To investigate population structure in European lobster, Jenkins et al. (2019a) developed a cost-efficient panel of 79 single nucleotide polymorphisms (SNPs). The same panel was subsequently applied by Ellis et al. (2020) to distinguish *H. gammarus*, *H. americanus*, and first-generation (F1) hybrids, although it was not originally designed as an ancestry-informative marker panel for hybrid detection. Because the SNPs were originally selected to capture genetic variation within *H. gammarus*, the panel may be subject to ascertainment bias when applied to interspecific hybrid detection. Such a marker set may contain loci that are informative for population structure within *H. gammarus* but less informative for distinguishing later-generation hybrid classes or weak introgression from standing variation within *H. gammarus*. Therefore, the extent to which this existing panel can resolve introgression over longer timescales, such as first- and second-generation backcrosses, requires explicit evaluation.

Here, we present an empirical resampling-based evaluation of this 79-SNP panel. Our aim is to evaluate the performance of this specific marker panel, rather than to provide any general benchmark of reduced SNP panels for hybrid detection. Using genotype resampling from empirical parental populations to simulate hybrid individuals with known ancestry proportions, we assess the panel's resolution in distinguishing not only pure species and F1 hybrids, but also first- and second-generation backcrosses. By simulating crosses directly from observed multilocus genotypes, we retained the allele-frequency distributions, missing-data patterns, and marker properties of the empirical reference dataset, a common strategy in validation studies of hybrid detection methods (Anderson and Thompson 2002; Vähä and Primmer 2006; Fitzpatrick 2012). Although our study is specific to *H. gammarus*, *H. americanus*, and the 79-SNP panel in question, our approach and results are an illustrative example of the challenges and possibilities of molecular introgression monitoring in applied management and conservation contexts.

## Materials and methods

### Empirical SNP dataset

All analyzes were based on an empirical SNP dataset comprising 1,591 individuals (Supplementary Table 1) genotyped at 79 SNP loci, including *H. gammarus*, *H. americanus*, and putative hybrids (Jenkins et al. 2019b; Jenkins et al. 2019c; Ellis et al. 2020). The American lobster parental reference comprised 38 *H. americanus* individuals, including 18 live-caught individuals (“Americanus”) and 20 cooked individuals (“AmerCook”). The European lobster parental reference comprised 1,265 Atlantic *H. gammarus* individuals, excluding putative hybrids. Mediterranean *H. gammarus* populations comprising 258 individuals were excluded from downstream resampling analysis because the target European lobster parental reference in this study was the Atlantic *H. gammarus* background relevant to documented *H. americanus* introductions and the empirical hybrid material. Including Mediterranean populations in the resampling framework would introduce within-species geographic structure within *H. gammarus*, potentially conflating the Mediterranean–Atlantic cline with interspecific ancestry differences between *H. gammarus* and *H. americanus*. Ellis et al. (2023) reported that the extension of the SNP panel did not substantially alter the observed clinal pattern of genetic variation, and the original marker set already captured the major axes of differentiation within European lobster. The empirical hybrid (“HybridX”) group comprised 30 individuals and was also excluded from parental resampling. Genotypes were handled in R v4.3.3 (R Core Team 2026) using the adegenet framework for genind objects (Jombart and Ahmed 2011). Empirical allele frequencies and genotype availability across reference groups are reported in Supplementary Table 2. Allele frequencies were calculated directly from unphased diploid genotype dosages within each reference group. For each SNP and group, the alternate allele frequency was calculated as the total alternate allele count divided by twice the number of individuals with nonmissing genotypes at that locus. Missing genotypes were excluded on a per-locus and per-group basis. No new sampling was conducted for this study, and no ethical approval was required.

### Linkage disequilibrium in the parental references

We characterized linkage disequilibrium (LD) in the parental reference samples to describe the empirical SNP panel, but not to test the robustness of the simulated classification results to LD. Because the resampling framework generates gametes by sampling alleles independently at each locus, the synthetic genotypes are expected to be in approximate linkage equilibrium regardless of LD present in the empirical references. Consequently, this framework cannot directly evaluate how empirical LD affects posterior assignment confidence in real multilocus genotypes.

To characterize LD in the empirical parental reference samples, we calculated pairwise genotype-dosage *r*^2^ separately within the American and Atlantic European parental references. Genotypes were coded as unphased allele dosages, where *x* denotes the alternate-allele dosage and $x\in \{\begin{array}{ccc} 0, & 1, & 2 \end{array}\}$, using the genotype matrix extracted from the adegenet genind object. For each pair of SNPs, *r*^2^ was calculated as the squared Pearson correlation between genotype dosages using individuals with nonmissing genotypes at both SNPs. Pairwise comparisons for which *r*^2^ could not be estimated because 1 or both SNPs had zero genotype variance, or because fewer than 3 individuals had nonmissing genotypes at both SNPs, were retained in the output table but treated as nonestimable. Correlations were calculated with the base R cor() function.

### Empirical resampling framework for generating hybrid classes

To evaluate whether the 79-SNP panel can distinguish pure individuals and hybrids of known ancestry proportions, we simulated synthetic genotypes by resampling from the empirical parental pools. This approach reconstructs expected genotype distributions under Mendelian segregation using observed allele-count data at each locus. For each locus, a haploid gamete was generated from a sampled diploid individual by sampling an allele with probability proportional to its observed allele count in that individual at that locus. Two gametes were then combined to create a diploid offspring genotype by summing the allele indicators across loci.

Using this procedure, we generated 7 “true” ancestry classes corresponding to expected proportions of *H. americanus* ancestry:

- 0: Atlantic European parental class (resampled European individuals).
- 1: American parental class (resampled American individuals).
- 0.5 (F1): offspring of 1 European gamete and 1 American gamete.
- 0.25, 0.125: backcrosses toward Europe (F1 × Europe; then BC1 × Europe).
- 0.75, 0.875: backcrosses toward America (F1 × America; then BC1 × America).

For each class, we generated balanced datasets with equal sample size (*N*) per class, using a grid of $N\in \{\begin{array}{ccccc} 20, & 50, & 100, & 200, & 500 \end{array}\}$ . For each *N*, we ran 100 independent replicates (each replicate using a unique random seed drawn from 1 to 100), regenerating all 7 classes in each replicate.

### Genetic assignment with snapclust

Genetic assignment was performed using snapclust (adegenet) with *k* = 2 parental clusters and hybrid modeling enabled (Jombart and Ahmed 2011;  Beugin et al. 2018). Because snapclust assigns arbitrary labels to the 2 parental clusters, we first matched the inferred parental clusters to the empirical parental reference groups and report them as Europe and America throughout. For each individual, the assigned class was defined as the snapclust class with the highest posterior probability after this mapping. Assignment accuracy was then calculated by comparing this assigned class with the expected class label defined a priori from the simulated ancestry coefficient, including the corresponding backcross direction.

Hybrid modeling was parameterized using a set of hybrid coefficients representing the expected ancestry proportions of the simulated genotypes. The hybrid coefficients were 0.125, 0.25, 0.5, 0.75, and 0.875. These were constructed by taking the base set (0.125, 0.25, and 0.5), which reflected first- and second-generation backcross expectations under simple Mendelian inheritance, and explicitly including complementary values (1−*x*) to ensure symmetric evaluation of backcrosses toward either parental cluster.

For each replicate dataset, snapclust returned posterior membership probabilities for the parental and hybrid classes. For evaluation, each individual was assigned to a single predicted class by selecting the class with maximum posterior probability. An individual was counted as correctly assigned if its predicted class label exactly matched the expected class label for its known (“true”) resampled class after mapping the inferred parental clusters to Europe and America. We refer to this throughout as “strict” classification or accuracy. Expected labels were defined for each true class (0, 0.125, 0.25, 0.5, 0.75, 0.875, 1), including the corresponding backcross direction.

### Reference-size sensitivity of assignments

To evaluate sensitivity to parental reference-size imbalance, we repeated the resampling and classification analysis after randomly subsampling the Atlantic European parental reference to match the American parental reference size (*n* = 38). In each replicate, 38 individuals were sampled without replacement from the Atlantic European parental pool, while the American parental reference was retained at its full size of 38 individuals. Synthetic genotypes were then generated using the same 7 ancestry classes, sampling depths, and replicate seeds as in the full-reference analysis. We compared the size-balanced analysis directly with the original full-reference analysis by true class, using both strict assignment accuracy and maximum posterior probability as summary measures.

### PCA and projection of resampled genotypes

We performed principal component analysis (PCA) on the empirical genotype matrix after mean-imputation of missing values and standardization (centering and scaling). The PCA loadings, centering, and scaling parameters were learned only from the empirical dataset. Resampled individuals of known ancestry were then projected into this fixed empirical PCA space by applying the same centering and scaling parameters and multiplying by the empirical PC loadings. As a result, empirical and resampled individuals share identical principal component axes, and differences in position reflect only their relative placement within the empirical genetic variation captured by the 79-SNP panel. The empirical hybrid (“HybridX”) individuals were included in this PCA space solely as a reference group; because they originate from a single family, they represent a restricted subset of hybrid genetic variation and are not intended to represent the full diversity of possible F1 hybrids.

### Statistical software

All computations were performed in R v4.3.3 (R Core Team 2026). Core dependencies for the resampling and snapclust pipeline included adegenet (Jombart and Ahmed 2011) and the tidyverse packages dplyr, tidyr, and tibble (Wickham et al. 2019). The analysis scripts and associated resources required to reproduce the pipeline are available in the project repository (see Data availability), together with the empirical data required to run the resampling.

### Use of large language model

We used GPT-5.2 via ChatGPT (OpenAI) to assist with code refactoring, language editing, and proofreading. All scientific interpretations, analyzes, and conclusions are the responsibility of the authors.

## Results

### Linkage disequilibrium in the parental reference

Genotype-dosage *r*^2^ values were generally low across the Atlantic European parental reference, with 10 of 3,081 SNP pairs exceeding *r*^2^ > 0.2. In the American reference, most SNP pairs were not estimable because 1 or both loci had zero variance or insufficient complete genotypes, reflecting the high frequency of fixed or nearly fixed loci in this reference group. The elevated-LD pairs were concentrated in a small number of loci. In the American reference, 4 estimable SNP pairs exceeded *r*^2^ > 0.2, involving 31967.C, 45217.G, 11071.G, and 55142.T. In the Atlantic European reference, 10 SNP pairs exceeded *r*^2^ > 0.2, all involving the 5 loci 65576.C, 65064.A, 6157.G, 53314.C, and 15128.C (Supplementary Table 3; Supplementary Fig. 1), while the distribution of estimable pairwise *r*^2^ values across the full panel is shown in Supplementary Fig. 2. Because the resampling procedure samples alleles independently across loci, this LD analysis should be interpreted as a descriptive characterization of the empirical reference panel rather than as a test of robustness of the simulated classification results to empirical LD.

### Classification accuracy across simulated hybrid classes and sampling depths

For all simulated hybrid classes, increasing the number of individuals sampled per class led to improvements in strict classification accuracy, with the most pronounced gains observed between 20 and 50 individuals per class. However, gains plateaued rapidly thereafter, with limited improvement beyond approximately 100 to 200 individuals sampled per class. This pattern was observed for both European- and American-biased backcrosses, with reduced discrimination among adjacent hybrid generations persisting across sampling depths (Fig. 1). Second-generation backcrosses exhibited the lowest strict accuracies across sampling depths, whereas first-generation backcrosses showed intermediate performance.

**Fig. 1.**
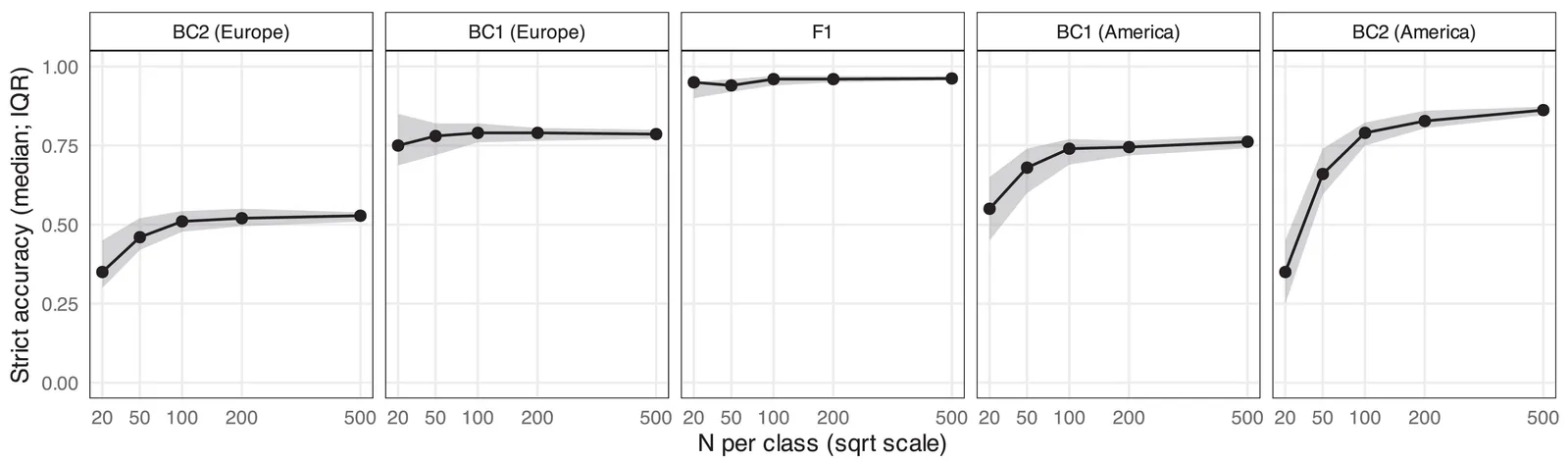
Strict classification accuracy across sampling depth for hybrid classes. Strict classification accuracy is shown as a function of the number of individuals sampled per class (*N* per class) across replicate resampling runs. Panels are faceted by the true resampled hybrid class: second-generation backcross to Europe (BC2 Europe), first-generation backcross to Europe (BC1 Europe), first-generation hybrid (F1), first-generation backcross to America (BC1 America), and second-generation backcross to America (BC2 America). Points and connecting lines indicate the median strict accuracy across replicates. Shaded ribbons represent the interquartile range (IQR), defined by the 25th and 75th percentiles (Q25 to Q75) of accuracy across replicates. The x-axis is shown on a square-root (sqrt) scale.

Subsampling the Atlantic European parental reference to match the American reference size (*n* = 38) produced classification patterns broadly consistent with the full-reference analysis (Supplementary Fig. 3). The size-balanced analysis showed a modest improvement in assignment of the European parental class, but the overall pattern of reduced resolution among European-biased backcross classes and neighboring hybrid classes remained consistent with the full-reference analysis.

### Projection of resampled hybrid genotypes onto empirical genetic space

Next, the principal component analysis (PCA) on the empirical genotype dataset characterized the major axes of empirical genetic variation among lobster groups for comparison against the simulated genotypes (Fig. 2). The first 2 principal components explained 7.6% and 5.4% of the total genetic variance, respectively. Atlantic European and American individuals formed well-separated clusters primarily along PC1. Empirical hybrids (HybridX) occupied intermediate positions between the Atlantic European and American clusters, consistent with admixed ancestry (Ellis et al. 2020). Because the empirical hybrid (“HybridX”) individuals originate from a single family group, their distribution reflects a restricted subset of hybrid genetic variation. A PCA including Mediterranean samples is shown in Supplementary Fig. 4. The Mediterranean populations were retained in the empirical dataset for geographic context and allele-frequency comparison, but were excluded from parental resampling pools because the resampling framework targeted the Atlantic European H. gammarus background relevant to the documented hybridization context.

**Fig. 2.**
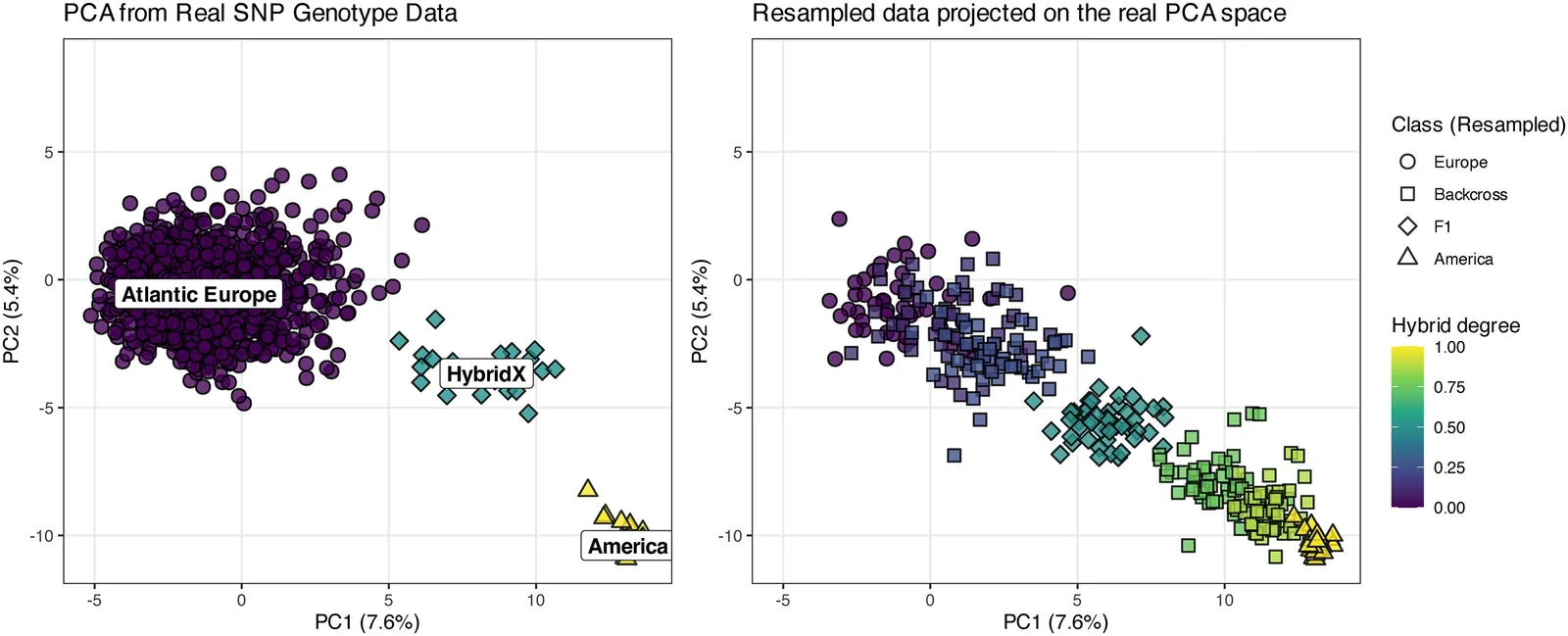
PCA of empirical and resampled lobster genotypes in a shared ordination space. The left panel shows the PCA based on the empirical SNP dataset (79 loci), illustrating geographic origins (Europe, HybridX, and America). The right panel shows the projection of resampled individuals (*N* = 50 individuals per class, seed = 1) onto the empirical PCA space. Both panels share identical axes and variance explained (PC1 = 7.6%, PC2 = 5.4%). In the right panel, color indicates the proportion of *H. americanus* ancestry (0 = Atlantic European parental, 1 = American parental), using the same color scale as in the empirical panel. Point shape denotes the true resampled class (Europe, backcross, F1, and America). Mediterranean individuals were used for PCA but were omitted from the visualization for clarity. The full PCA plot is shown as Supplementary Fig. 4.

Resampled genotypes of known ancestry were projected onto the empirical PCA space, using the same centering, scaling, and loading parameters derived from the empirical data. The resampled individuals formed a continuous gradient between the 2 parental clusters (Fig. 2). Individuals with ancestry proportions close to 0 or 1 overlapped closely with the corresponding Atlantic European and American empirical clusters, whereas intermediate hybrid classes (ancestry 0.25, 0.5, and 0.75) occupied positions between these parental extremes.

The ordering of resampled individuals along PC1 followed their ancestry proportions, indicating that the primary axis of empirical genetic differentiation largely reflects the ancestry gradient between the 2 parental lineages (Fig. 2). At the same time, intermediate hybrid classes showed substantial overlap in PCA space, particularly among neighboring ancestry categories. This overlap mirrors the distribution of snapclust assignments, where incorrect classifications occurred primarily between adjacent hybrid classes. Resampled hybrids overlapped with empirical hybrids (“HybridX”) in PCA space, although we reiterate: the latter should be interpreted only as a reference and not as representative of the full range of possible hybrid genotypes.

### Misclassification structure among hybrid classes

To explore the structure of classification errors in more detail, we visualized snapclust assignments for a representative resampled dataset with *N* = 500 individuals per class (seed = 1) as a confusion matrix (Fig. 3). Correct assignments were concentrated along the diagonal of the matrix, but the 2 parental classes differed in assignment stability. In the representative dataset shown in Fig. 3, true American parental individuals were assigned to the American parental class with a proportion of 1.00. By contrast, true European parental individuals were assigned to the European parental class with a proportion of 0.66, while 0.29 were assigned to BC2-Europe and 0.04 to BC1-Europe. Thus, although assignments to distant ancestry classes were rare, about 1 in 3 individuals from the European parental class were assigned to European-backcross categories.

**Fig. 3.**
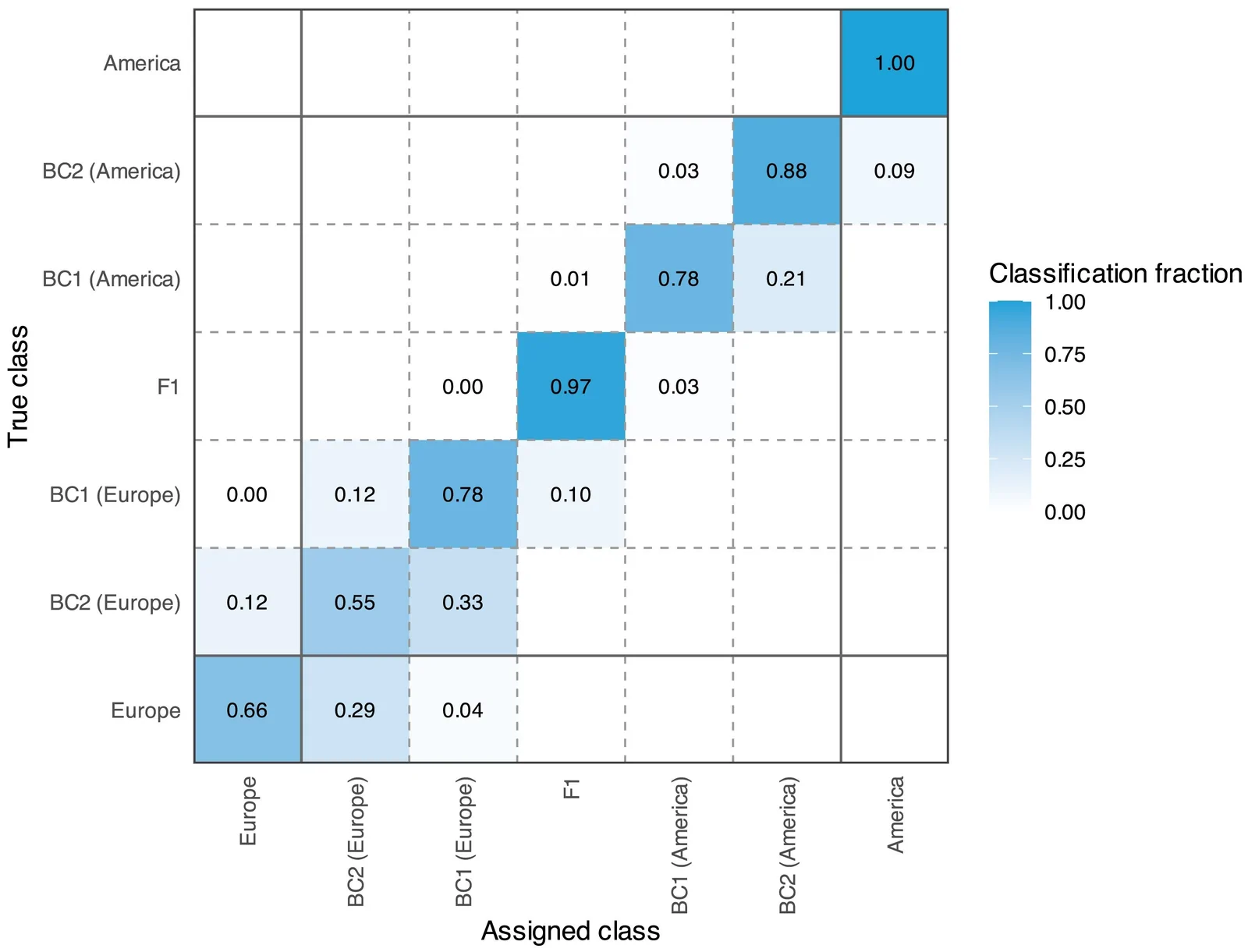
Misclassification structure among resampled ancestry classes. Snapclust assignments are shown for a representative resampled dataset with *N* = 500 individuals per class (seed = 1). Rows indicate the true resampled ancestry class and columns indicate the assigned class. Values show the fraction of individuals in each true class assigned to each inferred class, with rows summing to 1. Darker shading indicates a higher classification fraction.

### Calibration of posterior probabilities from snapclust

To finally assess whether posterior probabilities produced by snapclust reflect assignment reliability, we examined the relationship between maximum posterior probability and strict classification accuracy using a representative resampled dataset (Fig. 4). Individuals were grouped into bins according to their maximum posterior probability, and strict accuracy was calculated within each bin.

**Fig. 4.**
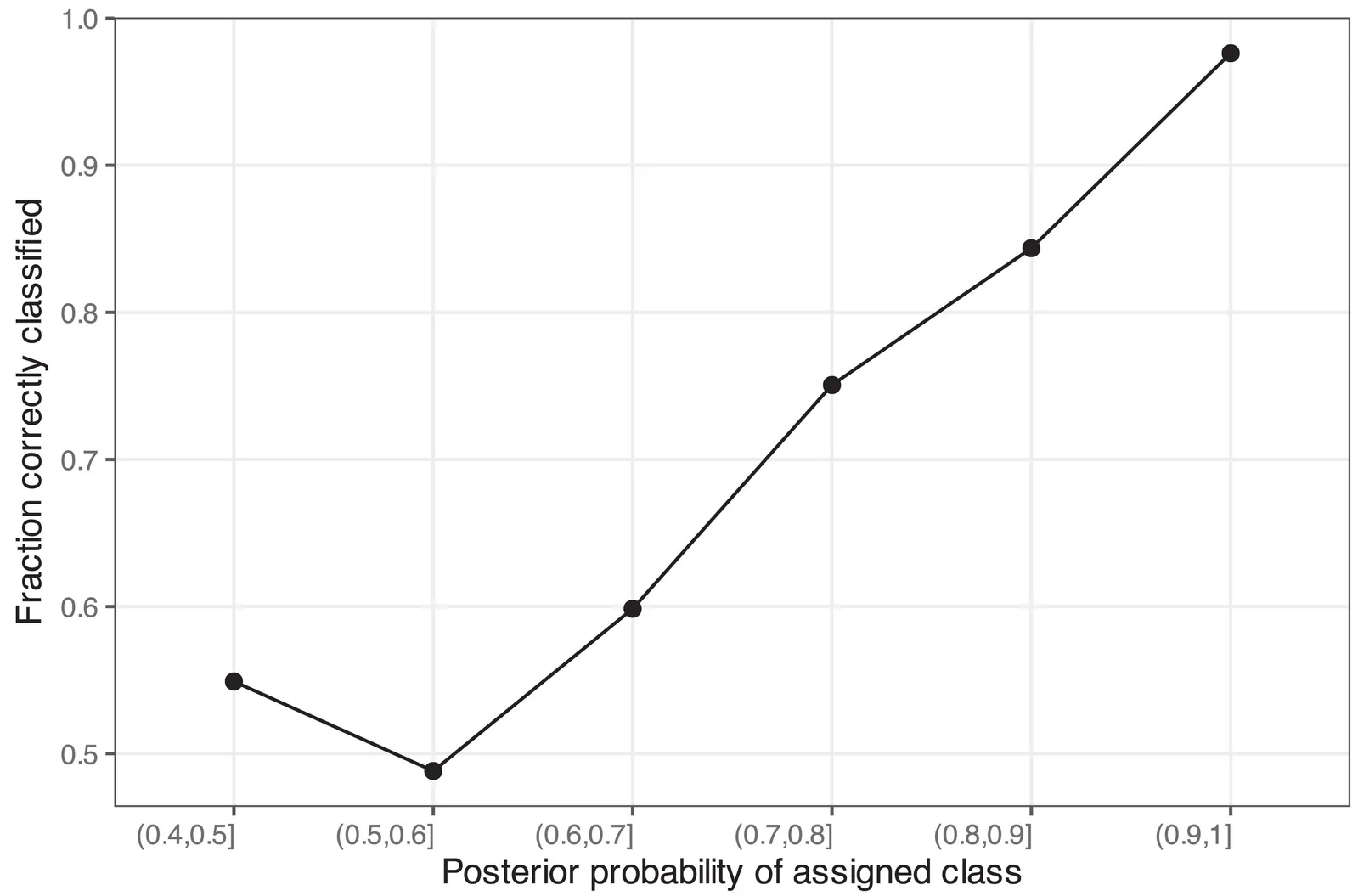
Relationship between snapclust posterior probabilities and classification accuracy. Strict assignment accuracy is shown as a function of the maximum posterior probability of the assigned class, using the same representative resampled dataset as in Fig. 3 (*N* = 500 individuals per class, seed = 1). Individuals were grouped into posterior probability intervals. Interval notation is used such that intervals of the form (*a*, *b*] include values >*a* and ≤*b*. For each posterior-probability interval, accuracy was calculated as the fraction of individuals whose assigned class exactly matched the true resampled class.

Strict accuracy increased monotonically with posterior probability. Assignments with posterior probabilities above 0.9 were associated with near-perfect strict accuracy, whereas assignments with lower posterior support showed substantially reduced accuracy. Individuals with posterior probabilities below 0.6 exhibited strict accuracies near 0.5, indicating considerable uncertainty in exact class assignment. These posterior probabilities should be interpreted as measures of confidence conditional on the model and marker set, rather than as absolute indicators of correct ancestry assignment.

## Discussion

### Ancestry resolution performance of the 79-SNP panel

Our results show that the 79-SNP panel captures broad differentiation between *H. gammarus and H. americanus.* However, classification accuracy declines for intermediate hybrid categories, especially at lower sample size. The size-balanced reference analysis showed broadly similar assignment patterns and posterior probabilities to the full-reference analysis, indicating that the main qualitative conclusions were not primarily driven by parental reference-size imbalance. The decline in strict accuracy for intermediate classes is consistent with theoretical expectations for hybrid classification based on multilocus markers (Vähä and Primmer 2006), although in the present study it is also shaped by the allele-frequency structure of the panel, including shared polymorphism between parental groups and the absence of species-diagnostic markers.

Because the panel was originally developed to capture population structure within *H. gammarus* (Jenkins et al. 2019b; Ellis et al. 2020), it is subject to ascertainment bias when applied to interspecific hybrid detection, which further contributes to ambiguity in ancestry assignment. As the signature of introgressed ancestry becomes subtle, adjacent hybrid classes occupy overlapping regions of genetic space and are inherently difficult to distinguish (Baack and Rieseberg 2007). Consequently, while the 79-SNP panel can identify broad ancestry classes and early-generation hybrids, strict separation among multiple adjacent hybrid classes is less certain given both the number of loci and their underlying allele-frequency distributions (Supplementary Table 2).

This issue is particularly important for parental class assignments. While resampled American parental individuals were assigned stably to the American class, approximately one-third of resampled Atlantic European parental individuals were assigned to European-backcross classes. This pattern is distinct from the expected ambiguity among neighboring hybrid classes. While misclassification among BC1, BC2, and F1 classes reflects overlap along a genuine ancestry continuum, assignment of resampled European parental individuals to backcross classes represents false-positive signals of introgression in native *H. gammarus* genotypes. The marked asymmetry between the European and American parental classes indicates that this behavior is not simply a generic consequence of using a reduced marker panel, but is related to the allele-frequency structure of this specific panel and the greater polymorphism retained within *H. gammarus*. Given the high false-positive rate, European-backcross assignments cannot be interpreted uncritically as evidence of *H. americanus*-introgression into *H. gammarus*, particularly when posterior support is low.

The patterns of uncertainty observed in strict classification were mirrored by projections of resampled genotypes onto the principal component space defined by the empirical dataset. Resampled individuals formed a smooth gradient between parental clusters, with substantial overlap among neighboring hybrid categories. Moreover, posterior probabilities from snapclust were strongly associated with strict classification accuracy: high posterior support corresponded to near-perfect strict assignment, while lower posterior probabilities indicated greater uncertainty. These posterior probabilities need to be interpreted as measures of confidence conditional on the model and marker set, rather than as absolute indicators of correct ancestry assignment. Still, probabilistic metrics are useful indicators of assignment confidence and can inform reporting thresholds and quantify uncertainty in applied contexts (Oliveira et al. 2015; Chakraborty and Rannala 2023; Zbinden et al. 2023).

### Implications for monitoring applications and future directions

Benchmarks of marker panels provide an assessment of performance under conditions that approximate natural genotype distributions. Our simulation design and results should be interpreted as a panel-specific benchmark rather than a general validation of reduced SNP panels for hybrid classification. The balanced class sizes we used in the resampling simulations were an analytical design for evaluating class-specific assignment performance, not an expected representation of applied screening conditions. In the wild, parental individuals are likely much more frequent than hybrids. The expected prevalence of hybrid classes should affect the practical interpretation of any assignment result. Moreover, our LD analysis should be interpreted only as a descriptive characterization of the empirical marker panel. Because the resampling framework samples alleles independently across loci, it cannot assess how empirical LD would affect posterior assignment confidence in real genotypes. Future work using linkage-aware simulations or higher-density genomic data could evaluate how empirical LD affects posterior assignment confidence, particularly for later-generation hybrid classes. It is also worth reiterating that the panel evaluated here was not originally designed to include, and does not include, species-diagnostic loci.

From a fisheries management perspective, the 79-SNP panel (Jenkins et al. 2019b; Ellis et al. 2020) could be a practical tool to rapidly monitor for broad ancestry categories when genomic resources are not available. In particular, we confirm that the panel can distinguish parental classes and F1 hybrids in regions where American lobsters have been recently introduced into European waters (Ellis et al. 2020). Such applications are relevant for biosecurity efforts to limit introgression into native European lobster populations. However, European-backcross assignments must be interpreted with great caution and should not generally be treated as evidence of introgression from *H. americanus*. This is because the panel assigned one-third of native *H. gammarus* genotypes to the BC1 and BC2 introgressed classes as false positives. Where precise ancestry resolution is required, higher-density genomic approaches are likely necessary.

## Supplementary Material

jkag206_Supplementary_Data

## Data Availability

This study used both empirical and resampling-based genotype data. The empirical genotype data, SNP information, simulation and analysis scripts, and code used to generate the figures and results are provided in Supplementary File 1. The code is also available at: https://github.com/mariesaitou/paper_2025-/tree/main/lobster_resampling. Empirical SNP genotype data were originally published by Ellis et al. (2020) and are included in the project repository with permission for noncommercial research use. No new raw sequence data were generated in this study. Supplemental material available at G3 online.
