# Supplementary material for: Resampling-based evaluation of a 79-SNP panel for hybrid classification across generations: a case study in European lobster (Homarus gammarus)
Source: G3 (Bethesda). 2026 Jul 29;16(10):jkag206. doi: 10.1093/g3journal/jkag206 (PMC13643775; doi:10.1093/g3journal/jkag206)
Supplement: Supplementary file 1 [file jkag206_supplementary_data.zip › Supplementary_Figures.docx]

**Figure S1. Pairwise genotype-dosage linkage disequilibrium among SNPs in the parental reference samples.** Heatmaps show pairwise r² values calculated separately within the American and Atlantic European parental reference samples. Only loci involved in at least one estimable pairwise comparison with r² > 0.2 in either parental reference are displayed. These comprise 9 loci in total. The remaining 70 loci had no estimable pairwise r² > 0.2 comparison in either parental reference and are omitted from this threshold-focused heatmap for readability. Blue shading indicates estimable r² values, with darker blue indicating higher r². Grey tiles indicate pairwise comparisons for which r² was not estimable because one or both loci had zero variance or insufficient complete genotypes within that reference.

**Figure S2. Distribution of pairwise genotype-dosage r² values across the full 79-SNP panel.** Histograms show the distribution of estimable pairwise r² values calculated separately within the American and Atlantic European parental reference samples. For each reference, all 3,081 possible SNP pairs were evaluated. In the American reference, 58 pairs were estimable and 3,023 were not estimable because one or both loci had zero variance or insufficient complete genotypes; 4 estimable pairs exceeded r² > 0.2. In the European reference, all 3,081 pairs were estimable, and 10 pairs exceeded r² > 0.2.

**Figure S3. Direct comparison of full-reference and size-balanced reference analyses. Class-specific strict assignment accuracy (A) and maximum posterior probability (B)** are compared between the original full-reference analysis and the analysis in which the Atlantic European parental reference was randomly subsampled to match the American parental reference size (n = 38). Panels show results across N = 20, 50, 100, 200, and 500 individuals per class. Points and lines show median values across replicate runs, and ribbons show the interquartile range.

**Figure S4. PCA of empirical and resampled lobster genotypes in a shared ordination space.**

The left panel shows the PCA result based on the empirical SNP dataset (79 loci), illustrating geographic origins (Europe, Mediterranean, HybridX, America). The right panel shows a projection of resampled individuals (a dataset with N = 50 individuals per class (seed = 1)) onto the empirical PCA space, and both panels share identical PC axes and variance explained (PC1 = 7.6%, PC2 = 5.4%). In the right panel, color gradient indicates the proportion of *H. americanus* ancestry (dark to light; 0 = European parental, 1 = American parental), with the sam color mapping applied to both left and right panels, while point shape in the projected panel denotes the true resampled class (Europe, backcross, F1 and America); Mediterranean individuals occur only in the empirical dataset for reference.
